# Post-mortem ventricular cerebrospinal fluid cell-free-mtDNA in neurodegenerative disease

**DOI:** 10.1038/s41598-020-72190-5

**Published:** 2020-09-17

**Authors:** Hannah Lowes, Marzena Kurzawa-Akanbi, Angela Pyle, Gavin Hudson

**Affiliations:** 1 Biosciences Institute, 4th Floor Cookson Building, Medical School, Newcastle University, Newcastle upon Tyne, NE2 4HH UK; 2 Clinical and Translational Research Institute, Newcastle University, Newcastle upon Tyne, NE2 4HH UK; 3 Wellcome Centre for Mitochondrial Research, Newcastle University, Newcastle upon Tyne, NE2 4HH UK

**Keywords:** Neurodegeneration, Neurodegenerative diseases

## Abstract

Cell-free mitochondrial DNA (cfmtDNA) is detectable in almost all human body fluids and has been associated with the onset and progression of several complex traits. In-life assessments indicate that reduced cfmtDNA is a feature of neurodegenerative diseases such as Parkinson’s disease, Alzheimer’s disease and multiple sclerosis. However, whether this feature is conserved across all neurodegenerative diseases and how it relates to the neurodegenerative processes remains unclear. In this study, we assessed the levels of ventricular cerebrospinal fluid-cfmtDNA (vCSF-cfmtDNA) in a diverse group of neurodegenerative diseases (NDDs) to determine if the in-life observations of reduced cfmtDNA seen in lumbar CSF translated to the post-mortem ventricular CSF. To investigate further, we compared vCSF-cfmtDNA levels to known protein markers of neurodegeneration, synaptic vesicles and mitochondrial integrity. Our data indicate that reduced vCSF-cfmtDNA is a feature specific to Parkinson’s and appears consistent throughout the disease course. Interestingly, we observed increased vCSF-cfmtDNA in the more neuropathologically severe NDD cases, but no association to protein markers of neurodegeneration, suggesting that vCSF-cfmtDNA release is more complex than mere cellular debris produced following neuronal death. We conclude that vCSF-cfmtDNA is reduced in PD, but not other NDDs, and appears to correlate to pathology. Although its utility as a prognostic biomarker is limited, our data indicate that higher levels of vCSF-cfmtDNA is associated with more severe clinical presentations; suggesting that it is associated with the neurodegenerative process. However, as vCSF-cfmtDNA does not appear to correlate to established indicators of neurodegeneration or indeed indicators of mitochondrial mass, further work to elucidate its exact role is needed.

## Introduction

Neurodegenerative disease (NDD) is an umbrella term that can be used to describe a diverse group of incurable nervous system disorders characterised by progressive loss of neuronal function and an overlapping spectrum of clinical phenotypes. The specific aetiology of many NDDs is complex and risk factors are multifactorial^1,2^, although there are often shared processes and pathological mechanisms^3^, such as the aggregation of misfolded proteins, proteotoxic stress and mitochondrial dysfunction^4^. NDDs can be difficult to diagnose and reliable biomarkers are needed to support early clinical diagnosis, to facilitate the delivery of effective treatments and improve patient welfare. Although some fluid-based biomarkers are established, for example, CSF biomarkers for Alzheimer’s disease (AD), biomarkers for other NDDs such as Parkinson’s disease (PD) have limited sensitivity and specificity^5^.

Circulating free nuclear DNA (cfDNA) was first identified in 1948^6^, but rose to prominence as a biomarker of cancer in the 1970s^7^. Since then, cfDNA levels have been linked to a diverse range of phenotypes including sepsis^8^, myocardial infarction^9^ and autoimmune disease^10^. More recently, research has demonstrated an association between cell-free-mitochondrial DNA (cfmtDNA) and several complex traits^11–23^, particularly neurological and neurodegenerative diseases such as PD^24,25^, AD^26,27^ and multiple sclerosis (MS)^14,28^. Its stability in extracellular fluids such as plasma, serum and cerebrospinal fluid has led many studies to suggest that cfmtDNA has utility as a biomarker of disease onset and progression.

However, there remain several unresolved issues. For example, there is no convincing biological explanation for the differential cfmtDNA levels seen in NDDs. Various hypotheses have been suggested, such as that cfmtDNA may be a product of either apoptotic or necrotic cell death^29,30^, the result of active expulsion during increased oxidative or metabolic stress, where it acts as a damage-associated molecular pattern molecule (DAMP)^31^, or a product of organellar trafficking between cells in a bid for cell viability^32^. Thus, it might be expected that cfmtDNA would be elevated in diseases that are associated with broad cell death and mitochondrial dysfunction. However, for NDDs at least, cfmtDNA is typically reduced compared to controls^24–28^. Further, few studies have shown correlations between cfmtDNA and the clinical and neurodegenerative hallmarks of disease. Whilst this does not necessarily diminish the usefulness of cfmtDNA as a biomarker, understanding the links between cfmtDNA and the neurodegenerative processes could improve our understanding of the pathology of NDDs. In the brain, cfmtDNA is likely to arise from the ependymal cells in the choroid plexus (CP), an area with abundant mitochondrial content^33^, specifically localised at the apical brush border^34^. Beyond AD^35^, the CP is not typically subject to gross neurodegeneration in NDD but does exhibit biological and pathophysiological changes associated with ageing and age-related disease^36^, including reduced CSF production and turnover, increased lipid (Biondi bodies) and protein (Aβ and α-synuclein) aggregates and reduced metabolic and enzymatic activity^35,36^. Furthermore, the CP appears to be particularly sensitive to mitochondrial dysfunction (a component of several NDDs^26,28,37^) commonly exhibiting physiological and anatomical changes in patients with Kearns-Sayre Syndrome^38^ and Leigh Syndrome^39^. Thus, although not directly implicated in NDD, the CP may also reflect disease-related changes within the CNS environment.

Here, to both expand upon the published observations in AD, PD and progressive MS and correlate cfmtDNA levels to established hallmarks of neurodegeneration, we assessed cfmtDNA levels in a large cohort of post-mortem ventricular CSF samples from a spectrum of NDDs.

## Results

### vCSF-cfmtDNA is reduced in PD, but not other NDDs

**Figure 1:**
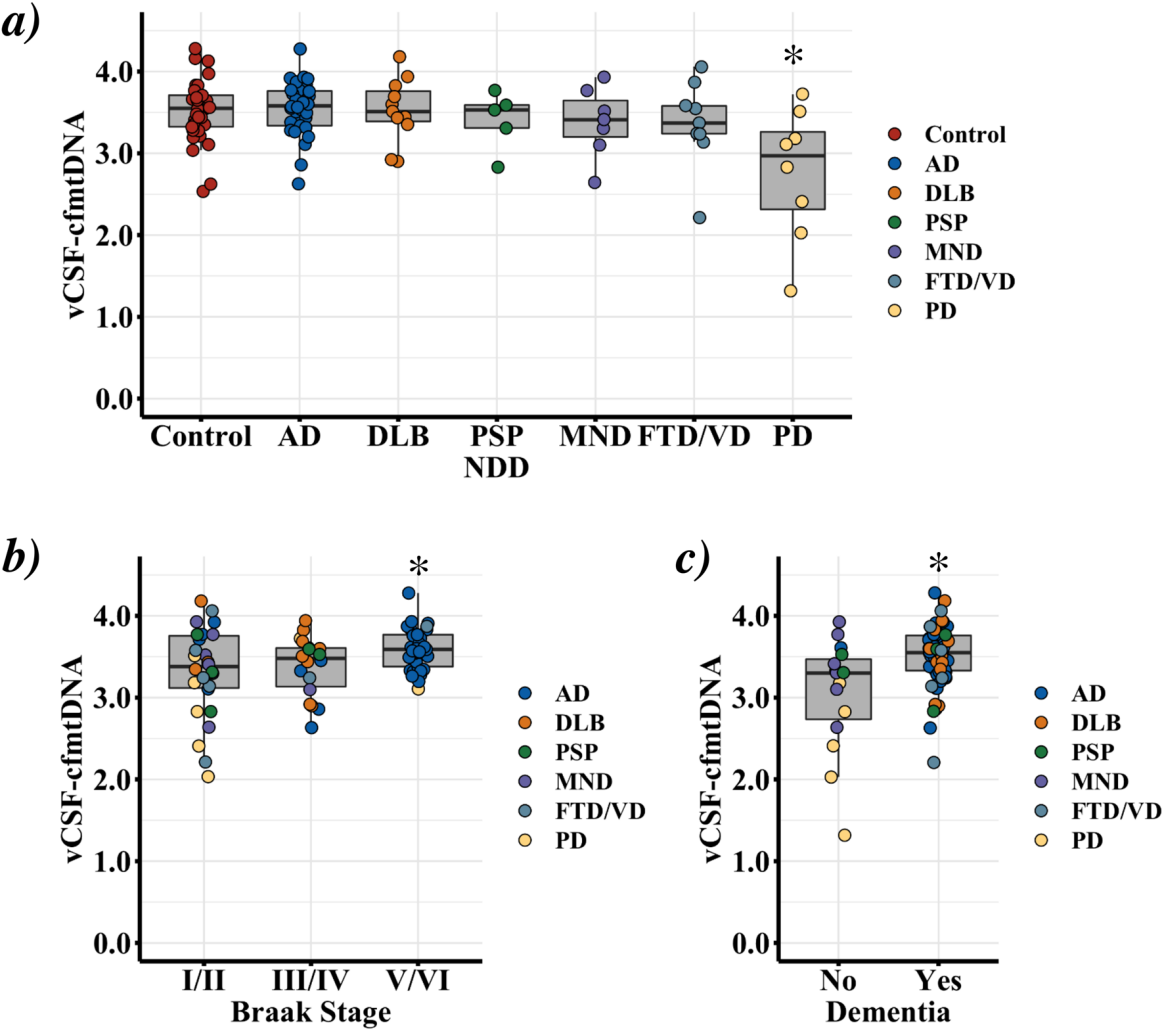
Comparison of vCSF-cfmtDNA levels in NDDs and matched controls. Boxplots of vCSF-cfmtDNA levels in NDD, showing vCSF-cfmtDNA levels in (**a**) each NDD and matched controls, (**b**) NDDs grouped and stratified by tau-Braak Stage and (**c**) NDDs stratified by dementia status. Where AD = Alzheimer’s Disease, DLB = dementia with Lewy bodies, PSP = progressive supranuclear palsy, MND = motor neuron disease, FTD/VD = frontotemporal lobar dementia/vascular dementia and PD = Parkinson’s disease. Boxes show median (thick line), 25th and 75th percentile and whiskers show 95% confidence interval. *indicates significance at *p* < 0.05 using ANOVA with Dunnett’s post-hoc test (**a**, with control as reference & **c**, with I/II as reference) and Student’s TTEST of yes versus no (b).

Similar to our previous cell-free mtDNA (cfmtDNA) work on lumbar CSF in PD^37^, we observed a significant reduction of ventricular CSF cfmtDNA (vCSF-cfmtDNA) in PD cases compared to controls (Fig. 1a, Dunnett’s p = 1.1 × 10^−4^). However, we observed no significant difference in vCSF-cfmtDNA levels between any other NDD group and controls (Dunnett’s *p* > 0.05, Fig. 1a) or when NDD patients were grouped and compared to controls (SFigure 1, TTEST *p* > 0.05). We also found no correlation between age of onset or disease duration and vCSF-cfmtDNA (r2 < 0.2, *p* < 0.05) (SFigures 2 and 3). We found no correlation between vCSF-cfmtDNA and postmortem delay when analysed as groups seperately or when combined.

### vCSF-cfmtDNA is increased in patients exhibiting neocortical pathology

To assess the links between vCSF-cfmtDNA and neurodegenerative disease, we next compared vCSF-cfmtDNA levels to NDD cases stratified by tau pathology Braak staging^40,41^. We observed a significant correlation between Braak stage (I to VI) and vCSF-cfmtDNA levels (*p* = 0.028, Pearson’s correlation = 0.31). To simultaneously increase statistical power, and perform a focused regional analysis, we grouped our analysis into three neuropathological regions; early entorhinal (Braak I/II), a limbic (Braak III/IV), and a late neocortical (Braak V/VI) stage^40,41^. This stratification indicated patients with neocortical pathology showed significantly higher vCSF-cfmtDNA than patients with stage I/II pathology (Dunnett’s *p* < 0.05, Fig. 1b). It should be noted that this effect appears to be driven by AD patients (31/33 or 94% of individuals with Braak V/VI were AD).

### vCSF-cfmtDNA is increased in patients with dementia

Around 80% of NDD patients (n = 65) were diagnosed with dementia before death. Similar to studies in sporadic Creutzfeldt-Jakob disease^42^ and AD dementia^27^, we observed significantly higher vCSF-cfmtDNA levels in patients diagnosed with dementia when compared to non-demented cases (TTEST *p* = 0.024, Fig. 1c). Unlike the elevation of vCSF-cfmtDNA seen in Braak V/VI, where the subset of samples were largely AD cases, demented cases were a cross-section of the NDD cohort (38 AD, 11 DLB, 9 FTD/VD, 3 PD, 3 PSP and 1 MND), indicating that this result is somewhat independent of regional tau pathology.

### Neurodegenerative protein markers associate with NDD but do not correlate to vCSF-cfmtDNA levels

To further assess the links between vCSF-cfmtDNA and neurodegeneration we compared cfmtDNA levels to the abundance of CSF protein markers of neurodegeneration^43,44^, including neuron-specific enolase (NSE)^45,46^, 14–3-3 zeta and 14–3-3 beta^47^, alpha-synuclein^48,49^ and tropomyosin receptor kinase B (TRKB)^50,51^. To investigate potential associations with mitochondrial debris, extracellular and/or synaptic vesicles, vCSF-cfmtDNA was compared to the levels of the most abundant synaptic vesicle membrane protein synaptophysin^52–54^ and mitochondrial markers: SDHA (an inner mitochondrial membrane marker)^55^, TFAM (a mitochondrial transcription factor, the expression of which often reflects mtDNA level)^56^ and porin (to investigate mitochondrial mass)^57^.

We compared protein levels between each NDD and controls, revealing significant associations between 14–3-3 zeta protein and AD, DLB and FTD/VD cases, synaptophysin protein and PD, DLB, FTD/VD and PSP cases, TFAM protein and DLB and FTD/VD cases and 14–3-3 beta and TRKB proteins with PD cases (STable 1a). However, 95% confidence intervals showed great variability within disease groups which may be reflective of modest sample sizes. Thus, to improve statistical power, we combined all NDDs into one group and compared the protein levels to controls (STable 1b).

**Figure 2:**
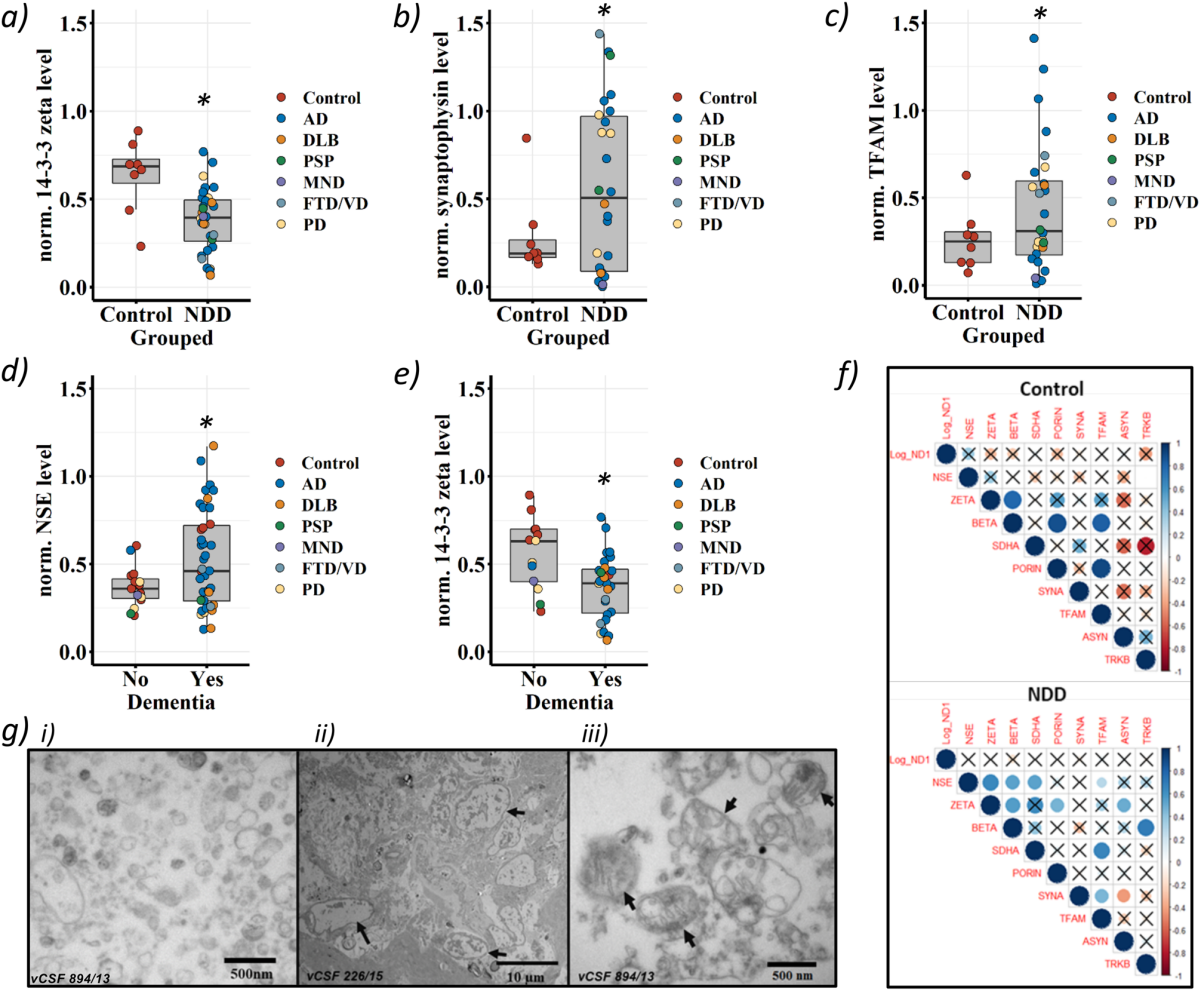
Comparisons of neurodegenerative protein markers and vCSF-cfmtDNA in NDDs and matched controls. Boxplots of vCSF protein levels, showing, (**a**) 14–3-3 zeta, (**b**) synaptophysin and (**c**) TFAM protein levels in combined NDDs and matched controls. (**d**) and (**e**) shows NSE and 14–3-3 zeta protein levels respectively in combined NDDs stratified by the in-life diagnosis of dementia. Boxes show median (thick line), 25th and 75th percentile and whiskers show 95% confidence interval. *indicates significance at *p* < 0.05 Student’s TTEST. (**f**) Correlograms of vCSF-cfmtDNA level and protein levels in controls (upper) and combined NDDs (lower). Where, colour intensity depicts r^2^ and X indicates *p* > 0.05, Log_ND1 = vCSF-cfmtDNA, NSE = neuron specific enolase, ZETA/BETA = 14–3-3 zeta/beta, SDHA = succinate dehydrogenase, SYNA = synaptophysin, TFAM = transcription factor A, mitochondrial, ASYN = alpha synuclein, TRKB = tropomyosin receptor kinase B. (**g**) SEM images of vCSF pellets, indicating: (i) vesicular-like structures, (ii) proteinaceous material and (ii-iii) potential mitochondria-like structures (black arrows).

Although generally, vCSF markers of neurodegeneration were reduced in combined NDD cases compared to controls (STable 1b), only 14–3-3 zeta reached statistical significance (TTEST *p* = 0.011, Fig. 2a and STable 1b). Next, we compared synaptophysin, a synaptic vesicle marker^58^, between combined NDD cases and controls. Synaptophysin was significantly elevated in NDD cases compared to controls (TTEST *p* = 0.001) (Fig. 2b and Stable 1b). Finally, we observed no significant association between SDHA or porin and NDD (STable 1b), although TFAM levels were elevated in NDD cases compared to controls (TTEST *p* = 0.006) (Fig. 2c and STable 1b).

Stratification of cases by the diagnosis of dementia revealed both a significant elevation of NSE (TTEST *p* = 0.038, Fig. 2d and STable 2a) and a significant reduction of 14–3-3 zeta in NDD cases with dementia when compared to NDD cases without dementia (TTEST *p* = 0.006, Fig. 2e and STable 2a). However, we observed no significant association between protein levels and Braak staging (STable 2b).

We did observe several novel interprotein correlations (Fig. 2f). Interestingly, the 14–3-3 proteins, zeta and beta, appear correlated (r2 > 0.5) in both NDD cases and controls. However, all other interprotein correlations appeared dependent on disease status. For example, NSE appears correlated (*p* < 0.05) to 14–3-3 zeta (r2 = 0.7), 14–3-3 beta (r^2^ = 0.5), SDHA (r^2^ = 0.5) and TFAM (r^2^ = 0.3) in NDD cases, but not controls. In NDD cases only, synaptophysin correlated (*p* < 0.05) to TFAM (r^2^ = 0.5) and alpha synuclein (r^2^ = -0.4), whereas in control only 14–3-3 beta correlated (*p* < 0.05) to porin (r^2^ = 0.9) and TFAM (r^2^ = 0.8). Whilst mitochondrial proteins SDHA and TFAM (r^2^ = 0.7, *p* < 0.05) correlated in NDD cases, porin and TFAM (r^2^ = 0.9, *p* < 0.05) correlated in controls.

Although several proteins were associated with NDD and dementia, we found no significant correlations between vCSF-cfmtDNA and protein levels in either combined NDD cases or controls (Fig. 2f), when NDDs were separated (STable 3), or when individuals were grouped by dementia status (STable 3).

### Electron microscopy of the vCSF indicates extracellular mitochondria-like structures

Recent work has suggested that, in addition to cfmtDNA, intact mitochondria can be observed in extracellular fluids such as plasma^59^. To investigate this in vCSF, we performed transmission electron microscopy (TEM) of two vCSF-cfmtDNA samples with large volumes of vCSF available (> 500 µl). TEM indicated the presence of abundant proteinaceous material (Fig. 2g) and various small and larger vesicular structures, potentially microvesicles, (Fig. 2gi), but no cellular debris. Cell-free mitochondria-like structures could be detected with double membranes and remnants of cristae like morphology, however, these were scarce and showed gross morphological changes and partial membrane degradation (Fig. 2gii-iii).

### vCSF-cfmtDNA circulates as intact genomes, with low deletion and heteroplasmy levels

We next assessed the integrity of vCSF-cfmtDNA using both qPCR, to measure mtDNA deletion levels^28^, and next-generation-sequencing (NGS), to assess mtDNA mutational load^28^.

**Figure 3:**
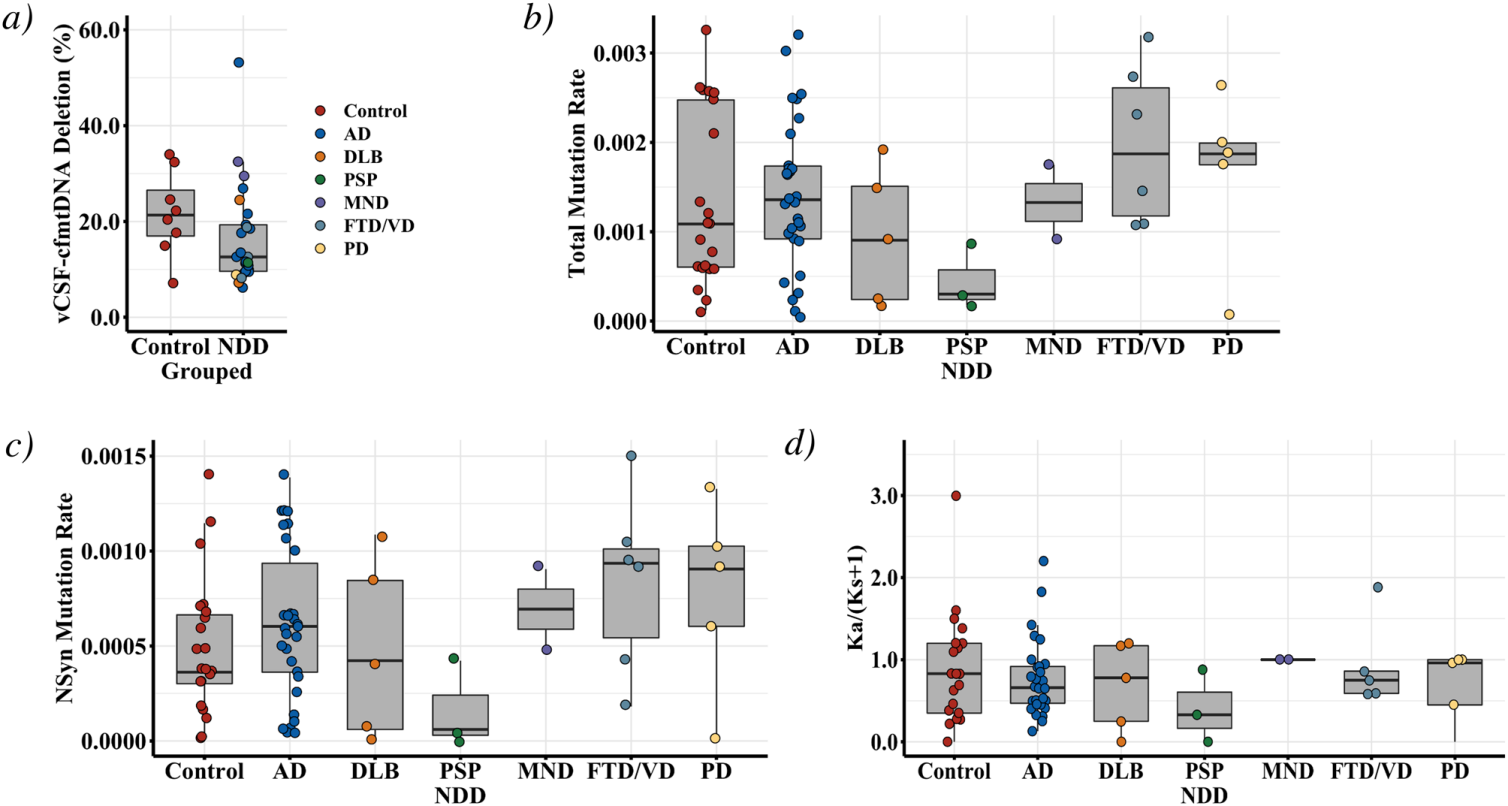
Analysis on vCSF-cfmtDNA integrity in NDDs and matched controls. Boxplots of vCSF-cfmtDNA integrity in NDD, showing (**a**) vCSF-cfmtDNA deletion level in NDDs and matched controls, (**b**) the comparative total vCSF-cfmtDNA heteroplasmic mutation rate in each NDD and matched controls, (**c**) the comparative non-synonymous vCSF-cfmtDNA heteroplasmic mutation rate in each NDD and matched controls and (**d**) the vCSF-cfmtDNA Ka/Ks(+ 1) for in each NDD and matched controls. Where AD = Alzheimer’s Disease, DLB = dementia with Lewy bodies, PSP = progressive supranuclear palsy, MND = motor neuron disease, FTD/VD = frontotemporal lobar dementia/vascular dementia and PD = Parkinson’s disease. Boxes show median, 25th and 75th percentile and whiskers show 95% confidence interval.

Only 31 samples (27%), 23 (29%) NDD and 8 controls (23%), harboured mtDNA deletions above the established detection threshold of 10%^60^, suggesting that in the majority of samples (73%) vCSF-cfmtDNA is intact and full length. Although mtDNA deletion levels appeared lower in NDD cases compared to controls (Fig. 3a) this did not reach statistical significance (TTEST *p* > 0.05).

Next, we performed NGS of vCSF-cfmtDNA from 72 samples (51 cases, or 71%, and 21 controls, 29%). Although we were able to detect heteroplasmic variants (average = 23.2 in NDD and 22.3 in controls), we observed no significant difference between NDDs and controls (Dunnett’s > 0.05) when analysed as total heteroplasmic mutation rate (Fig. 3b) or when comparing non-synonymous heteroplasmic (i.e. putatively functional) variation (Fig. 3c). Combining NDD cases, to improve statistical power, and comparing mutation rates to controls also failed to identify a significant difference (TTEST *p* > 0.05, SFigure 4a and b). In previous work^28^, we had observed an imbalance in the Ka/Ks ratio (an indication of variant selective bias) of vCSF-cfmtDNA between progressive MS patients and controls However, we observed no significant difference in the Ka/Ks ratio of vCSF-cfmtDNA in NDDs compared to controls when analysed individually (Fig. 3d, Dunnett’s > 0.05) or when grouped as NDD disease (SFigure 4c TTEST *p* > 0.05).

## Discussion

Several studies have associated cell-free mtDNA (cfmtDNA) with human disease^11–23^, particularly neurodegenerative disease (NDD)^26,28,37^. Whilst these studies were typically aimed at identifying biomarkers of disease, they were unable to link cfmtDNA levels to pathology. In this study, we measured cfmtDNA in post-mortem ventricular CSF (vCSF), correlating vCSF-cfmtDNA levels to established markers of neurodegeneration to determine if the reported in-life cfmtDNA measurements in lumbar CSF would translate through to vCSF mtDNA levels and neuropathological assessment.

Our results indicate that, in line with our earlier observations in lumbar CSF^25,61^, vCSF-cfmtDNA is reduced in PD compared to controls, and this phenomenon appears to be specific to PD cases, rather than a feature of other NDDs. However, our data suggest that the relationship between vCSF-cfmtDNA and NDD may be more complex, possibly reflecting the diverse and multifaceted underlying pathoetiology of each disease. Intriguingly, a subsequent analysis indicates that elevated vCSF-cfmtDNA is associated with the degree of neuropathology, with higher levels of vCSF-cfmtDNA associated with late neocortical tau aggregations (Braak stage 5/6) and dementia.

In PD, the reduced vCSF-cfmtDNA we observed suggests that reduced cfmtDNA persists throughout the disease course with post-mortem vCSF-cfmtDNA levels comparable to those we observed in early-stage PD lumbar CSF^25,37^. How this relates to the neurodegenerative process in PD remains still unclear as we found no correlation between vCSF-cfmtDNA and neurodegenerative protein markers. However, the answers may reside in the relationship between cfmtDNA and neuronal mtDNA copy number. Previous work has shown that neuronal mtDNA copy-number is reduced in PD^24,62^, particularly in dopaminergic neurons^63^, and is correlated to mitochondrial dysfunction^62^. Thus, the reduced vCSF-cfmtDNA in PD may reflect an already depleted cellular mtDNA pool, with PD simply having less mtDNA to release. Further, reduced vCSF-cfmtDNA in PD may be a reflection of the treatment that PD patients have received, as our recent analysis indicates that reduced vCSF-cfmtDNA in PD correlates to levodopa effective daily dose^25^. Thus, as treatment in the latter stages of PD typically moves from symptom relief and prevention of motor symptoms (i.e. L-dopa) towards non-motor treatments and palliative care^64^, it is possible that the reduction of vCSF-cfmtDNA occurs early in the disease course and, for the majority of PD cases, remains reduced. However, this can only be tested in a longitudinal study that allows in-life lumbar CSF-cfmtDNA levels to be correlated with post-mortem vCSF-cfmtDNA levels.

Unlike PD, we did not observe a significant reduction in vCSF-cfmtDNA in the other NDDs.

Previous literature^26^ suggests that ‘probable’ AD cases (defined as those with low CSF amyloid beta_1-42_ and elevated tau levels) and high-risk for AD individuals (defined as asymptomatic but with low CSF amyloid beta_1-42_) exhibit significantly lower lumbar CSF cfmtDNA than controls. Conversely, a study of well-characterised AD patients reports significantly higher lumbar CSF cfmtDNA level than that of cognitively healthy controls^27^.

In our study, AD vCSF-cfmtDNA levels were not significantly different from matched controls. However, we did observe increased vCSF-cfmtDNA in NDD cases who exhibited extensive neocortical involvement (Braak V/VI^40,41^ when compared to Braak stages I/II) and individuals who had progressed to dementia. These results are likely self-confirmatory as AD cases made up 93% and 59% of all cases from both groups respectively (31/33 Braak stage V/VI cases and 38/65 dementia cases). When taken together, this suggests that in AD cfmtDNA levels may rise with disease severity and neuropathology, beginning at markedly low levels in pre-clinical stages and rising to exceed that of controls when dementia becomes severely debilitating and there is widespread neocortical pathology. However, longitudinal studies in AD would be needed to confirm this.

Elevated cfmtDNA has been observed in patients with traumatic brain injury^65^, relapsing–remitting multiple sclerosis^14^ and Anti-NMDAR Encephalitis^66^, suggesting that elevated cfmtDNA may be a component of the inflammatory response^14,65,66^. The innate immune system is involved in the pathoetiology of many NDDs^67^ and can be activated by mtDNA. Circulating cfmtDNA is a damage-associated molecular pattern (DAMP) molecule that can trigger the antimicrobial and inflammatory response^68^ after shock, injury^68^, infection^69^ and cancer^70^. Thus, given the increased neuroinflammation observed in NDDs^71^, our data suggest that the increase in vCSF-cfmtDNA we observed in chronic NDD may be a component of this response and may be a component of the pathogenesis of NDD and future work should attempt to link cfmtDNA to CSF markers of neuroinflammation.

To investigate the links between cfmtDNA and neurodegeneration further, we compared vCSF-cfmtDNA levels to the levels of reported CSF protein markers of NDD. Similar to others^72^, we hypothesised that vCSF-cfmtDNA levels would correlate to indicators of neuronal cell death, which may, in turn, explain the origins of vCSF-cfmtDNA. However, although we observed associations between protein levels and disease, we did not observe associations between these proteins and vCSF-cfmtDNA. This suggests that vCSF-cfmtDNA levels are not wholly coupled to apoptosis or derived from cellular debris. It must be noted, however, that we chose these proteins based on a priori assumption regarding their role in disease pathogenesis or pathology. Thus, further work should utilise an unbiased proteomic approach (i.e. mass spectrometry), potentially establishing novel protein interactions and further developing the understanding of underlying mechanisms involved in this biological phenomenon.

Previous work in serum indicates that cfmtDNA may be derived from circulating intact mitochondria^59^ and work in C. Elegans identified mitochondria in extracellular vesicles extruded under neurotoxic stress^73^. Similarly, we were able to detect the presence of cell-free mitochondria-like structures within vCSF. However, unlike the ‘in-life’ observations of intact and functionally competent mitochondrial made by Al Amir Dache et al. (2020), the mitochondrial structures we identified in NDD vCSF were sparse, grossly degraded and lacking defined membranes and cristae shape. Given that mitochondrial dysfunction is a component of many NDDs^4,62,63^, mitochondrial expulsion may be linked to pre-existing dysfunction, but it is also possible that the degraded morphology is a result of the post-mortem nature of the vCSF. To investigate further, we measured the abundance of the mitochondrial proteins SHDA^55^, TFAM^56^ and porin^57^. Although these mitochondrial markers were detectable in both NDD and controls they did not correlate to vCSF-cfmtDNA, further suggesting vCSF-cfmtDNA levels are independent of cell-free mitochondrion abundance. TEM of post-mortem CSF suggested the presence of extracellular vesicles within the vCSF, in line with similar reports^74,75^, which may point towards a possibility of an active release of cellular mitochondria to CSF via the extracellular vesicles route. Elevated CSF synaptophysin a synaptic vesicle marker, has been observed in patients with traumatic brain inury^75^, and its release into CSF is associated with neuronal cytotoxicity^76^. Similarly, in our study synaptophysin was significantly enriched in vCSF of NDDs, however, we found no correlation between vCSF-cfmtDNA and synaptophysin protein levels. Taken together, these data suggest that further work, specifically focusing on vCSF extracellular vesicles and quantifying their ccf-mtDNA content, could be vital in elucidating the origin and release of ccf-mtDNA.

MtDNA lesions and mutations are a hallmark of many NDDs^77^, with high levels of mtDNA deletions and somatic (heteroplasmic) mutations identified in disease-specific brain regions of PD^78,79^ and AD^80^ patients. Similar to previous work^81^, we used qPCR and NGS to interrogate the integrity of vCSF-cfmtDNA under the hypothesis that increased vCSF-cfmtDNA release linked to mtDNA maintenance mechanism, where mutated mtDNA is expelled from cells to maintain a ‘wild-type’ mtDNA pool. Our analysis indicates that although mtDNA deletions and point mutations were present in NDD vCSF-cfmtDNA, they were not significantly more frequent than in controls. Whilst this does not necessarily negate the hypothesis that vCSF-cfmtDNA is a mechanism for maintaining a healthy cellular mtDNA pool, our results do suggest that this mechanism is independent of disease status. However, it must be noted that a potential reason for this could be neuronal ‘survivor bias’; where, due to the end-stage of disease, only the cells with low-level mtDNA defects remain. This is somewhat supported by brain mtDNA heteroplasmy analysis^82^, whereby post-mortem, end-stage NDDs and controls show similar brain mtDNA heteroplasmy.

Our work is not without limitations. Firstly, the availability of post-mortem vCSF samples is limited and future work should expand the cohort sizes, particularly of non-AD NDDs. Secondly, although we were able to identify several protein-NDD associations, we were unable to link vCSF-cfmtDNA to established hallmarks of neurodegeneration. Thus, future work should take a more unbiased proteomics approach, for example using mass-spectrometry to identify all proteins with the vCSF. Finally, as discussed, neuroinflammation is demonstrated to be a key feature of many NDDs, most likely contributing to the pathogenesis of these disorders (as reviewed in^83^). Interestingly, cfmtDNA is also implicated in the inflammatory process, and previous studies have shown direct interplay between levels of this inflammatory molecule and important inflammatory cytokines in both human plasma^84^ and CSF^66^. This is a particularly crucial area for future study as it may reveal functional attributes of the cfmtDNA and its potential role in the mediation of the immune response in NDD.

## Conclusions

We conclude that vCSF-cfmtDNA is reduced in PD, but not other NDDs. Although its utility as a prognostic biomarker is severely limited, our data indicate that higher levels of vCSF-cfmtDNA are associated with more severe clinical presentations; suggesting that it plays a role in the neurogenerative process. However, as vCSF-cfmtDNA does not appear to correlate to established indicators of neurodegeneration or indeed indicators of mitochondrial mass, further work to elucidate its exact role is needed.

## Methodology

### Ventricular CSF sample cohort

We used 115 ventricular cerebrospinal fluid (vCSF) samples, obtained from the Newcastle Brain Tissue Resource (NBTR) and the Imperial College Tissue Bank (80 Neurodegenerative disease cases: Alzheimer’s disease (AD) = 40, dementia with Lewy bodies (DLB) = 11, progressive supranuclear palsy (PSP) = 5, motor neurone disease (MND) = 7, frontal temporal/vascular dementia (FTD/VD), Parkinson’s disease (PD) = 8, and 35 controls). vCSF was collected post-mortem from hemisected brains and immediately stored at -80 °C in polypropylene cryovials, which do not influence biomarker outcome^85^. Samples were prepared via methods described previously^86,87^. Briefly, vCSF was centrifuged (2000 g for 10 min) at room temperature, aliquoted into CSF collection tubes and stored upright at − 80 °C. All cases were confirmed in life by a local clinician and re-assessed neuropathologically post-mortem. All controls were negative for hallmarks of neurodegeneration or inflammation.

### VCSF-cfmtDNA quantification

Circulating cell-free mitochondrial DNA was quantified by established triplex Taqman qPCR^28,60,87^. VCSF-cfmtDNA level was calculated as the absolute measurement of MTND1, derived from a triplicated standard curve and expressed as copies per 1 µl of CSF. mtDNA deletion level was expressed as a ratio of MTND1 to MTND4 as described previously^28,60,87^.

### VCSF protein analysis

Protein markers of neuronal damage (neuron specific enolase, NSE; 14–3-3 beta; 14–3-3 zeta; tropomyosin receptor kinase B, Trkb), the synaptic vesicle membrane (synaptophysin) and mitochondrial components (SDHA; Porin; Tfam), were measured in all samples with sufficient vCSF (42 NDD patients; minimum in each disease group = AD 18, DLB 4, MND 1, FTD/VD 2, PSP 2, PD 5 and 14 controls; minimum 8), using western blot. Protein levels were normalised to total protein level measured with BLOT-FastStain™. Alpha-synuclein expression was measured by ELISA (LEGEND MAX™ Human alpha-synuclein ELISA kit) in the same samples as above, as per the manufacturer’s instructions.

### VCSF transmission electron microscopy (TEM)

Transmission electron microscopy was performed on a subset of vCSF samples. Briefly, vCSF was pelleted, fixed, dehydrated and then embedded into Taab medium epoxy resin, as described previously^88^. After polymerisation, ultrathin Sects. (70 nm) were collected, stained with uranyl acetate and lead citrate and then imaged on a Philips CM100 Transmission Electron Microscope (TEM).

### vCSF-cfmtDNA sequencing and bioinformatic analysis

Deep sequencing was performed on vCSF-cfmtDNA extracts from a subset (where volume and mtDNA concentration permitted), of vCSF samples (53 NDD patients and 21 controls) as described previously^28,89^. Briefly, mtDNA was extracted from vCSF using Ultrapure™ and ethanol precipitation, as per manufacturers guidelines, and enriched using three amplicon long-range PCR, covering the entire mtDNA genome. PCR products were purified, pooled and prepared using Illumina Nextera XT DNA Library Preparation Kit. Sequencing was performed using the Illumina Miseq v3.0 platform in paired-end, 250 bp reads. Post-run FASTQ files were analysed using an established in-house bioinformatics pipeline, as described previously^28^.

### Statistical analysis

Normality of vCSF-cfmtDNA distributions were assessed by Shapiro-Wilks and could not be rejected at the 0.05 level. Thus, all vCSF-cfmtDNA levels are expressed as log [10] copy-number per microliter. Data were analysed in R (v4.0)^90^ using data appropriate tests (detailed in the text). Statistical significance was set at *p* < 0.05. All tests were two-tailed with α = 0.05. Correlograms of protein levels were created using corrplot (v0.84) in R.

### Ethics approval

Donors or next of kin provided informed consent to donate tissue and all procedures were approved by the local UK National Health Service Research Ethics Committee. All procedures were approved by each Local Research Ethics Committee and appropriate informed consent was obtained from donors or next of kin for sample donation. Newcastle Brain Tissue Resource has been approved as a Research Tissue Bank by NRES Committee North East (Ref. No 08/H0906/136 + 5) and Parkinson's UK Brain Bank at the Imperial College London has been approved as a Research Tissue Bank by the Wales Research Ethics Committee (Ref. No. 08/MRE09/31 + 5). All methods were carried out in accordance with relevant guidelines and regulations.

## Data availability

All data, genetic and proteomic, is available upon request.

## Supplementary information

Supplementary Tables.

Supplementary Figures.
