# Supplementary material for: Post-mortem ventricular cerebrospinal fluid cell-free-mtDNA in neurodegenerative disease
Source: Sci Rep. 2020 Sep 17;10:15253. doi: 10.1038/s41598-020-72190-5 (PMC7499424; doi:10.1038/s41598-020-72190-5)
Supplement: Supplementary file 2 — Supplementary Figures. [file 41598_2020_72190_MOESM2_ESM.docx]

***Title: Post-mortem ventricular cerebrospinal fluid cell-free-mtDNA in Neurodegenerative disease***

Author List:

Hannah Lowes PhD^1,3^,

Marzena Kurzawa-Akanbi PhD^1^,

Angela Pyle PhD^2,3^,

Gavin Hudson PhD^1,3^

Author Affiliations:

^1^Biosciences Institute, Newcastle University, Newcastle upon Tyne, NE2 4HH, UK.

^2^Clinical and Translational Research Institute, Newcastle University, Newcastle upon Tyne, NE2 4HH, UK.

^3^Wellcome Centre for Mitochondrial Research, Newcastle University, Newcastle upon Tyne, NE2 4HH, UK.

***Contents:***

***SFigure 1.*** *Boxplot of vCSF-cfmtDNA levels in all NDD cases combined****.***

***SFigure 2.*** *Scatter plot of NDDs age of onset against vCSF-cfmtDNA levels.*

***SFigure 3.*** *Scatter plot of NDDs disease duration against vCSF-cfmtDNA levels.*

***SFigure 4.*** *Boxplots of vCSF-cfmtDNA integrity in NDDs combined.*

***
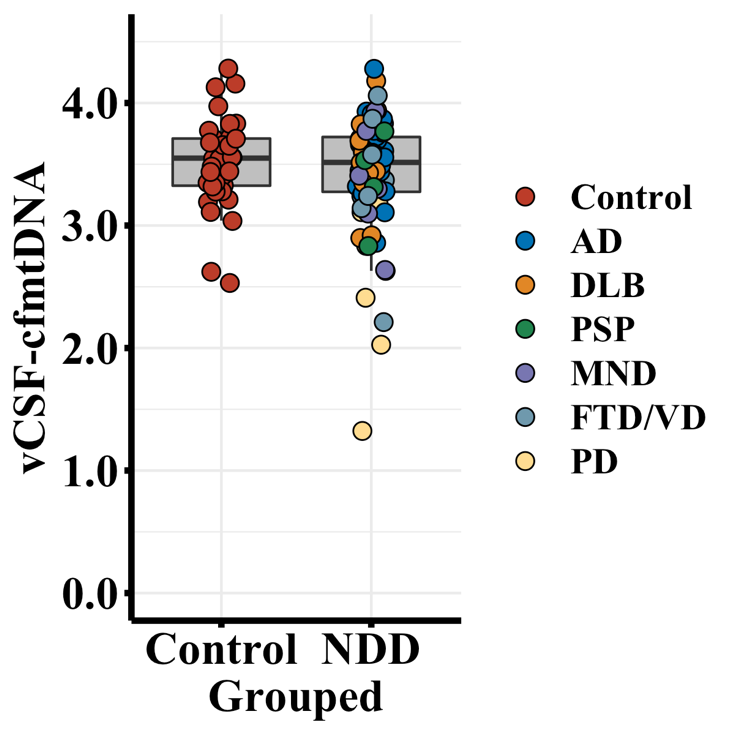
***

***SFigure 1.*** *Boxplot of vCSF-cfmtDNA levels in all NDD cases combined. Where, AD=Alzheimer’s Disease, DLB=dementia with Lewy bodies, PSP=progressive supranuclear palsy, MND=motor neuron disease, FTD/VD=frontotemporal lobar dementia/vascular dementia and PD=Parkinson’s disease. and matched controls. Boxes show median, 25^th^ and 75^th^ percentile and whiskers show 95% confidence interval. TTEST p>0.05.*

*
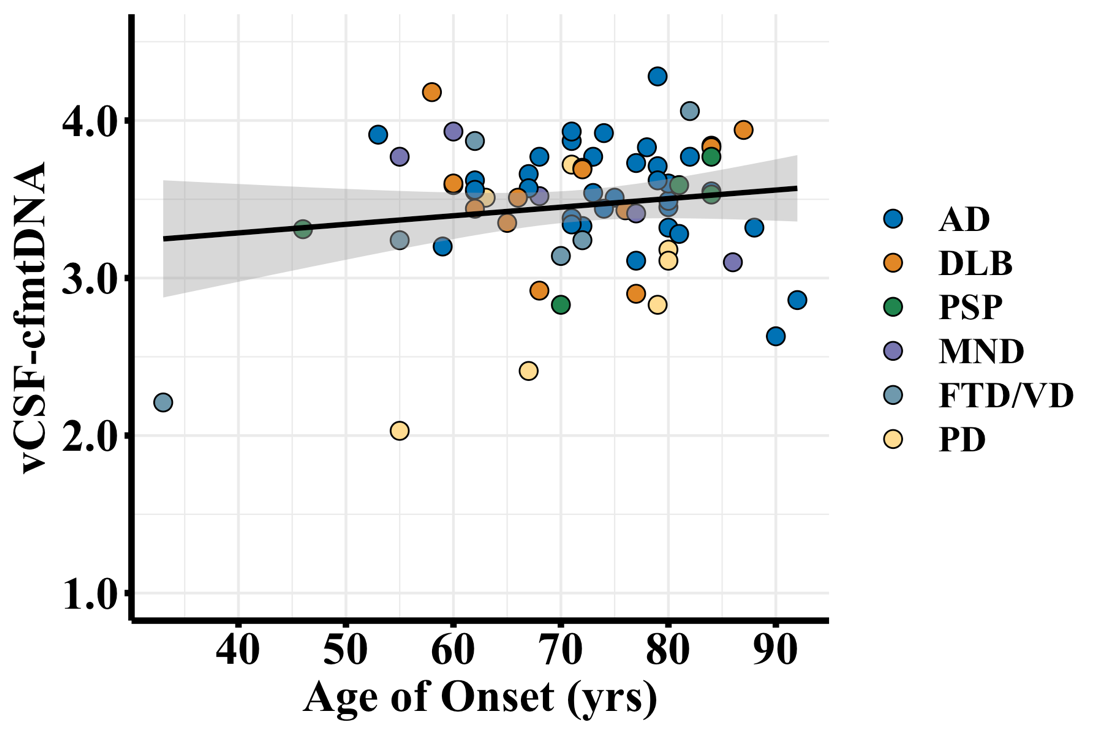
*

***SFigure 2.*** *Scatter plot of NDDs age of onset (x, years) against vCSF-cfmtDNA levels (y). Where, AD=Alzheimer’s Disease, DLB=dementia with Lewy bodies, PSP=progressive supranuclear palsy, MND=motor neuron disease, FTD/VD=frontotemporal lobar dementia/vascular dementia and PD=Parkinson’s disease. and matched controls. Line is linear regression line (r2=<0.2) with 95% confidence interval, p>0.05.*

*
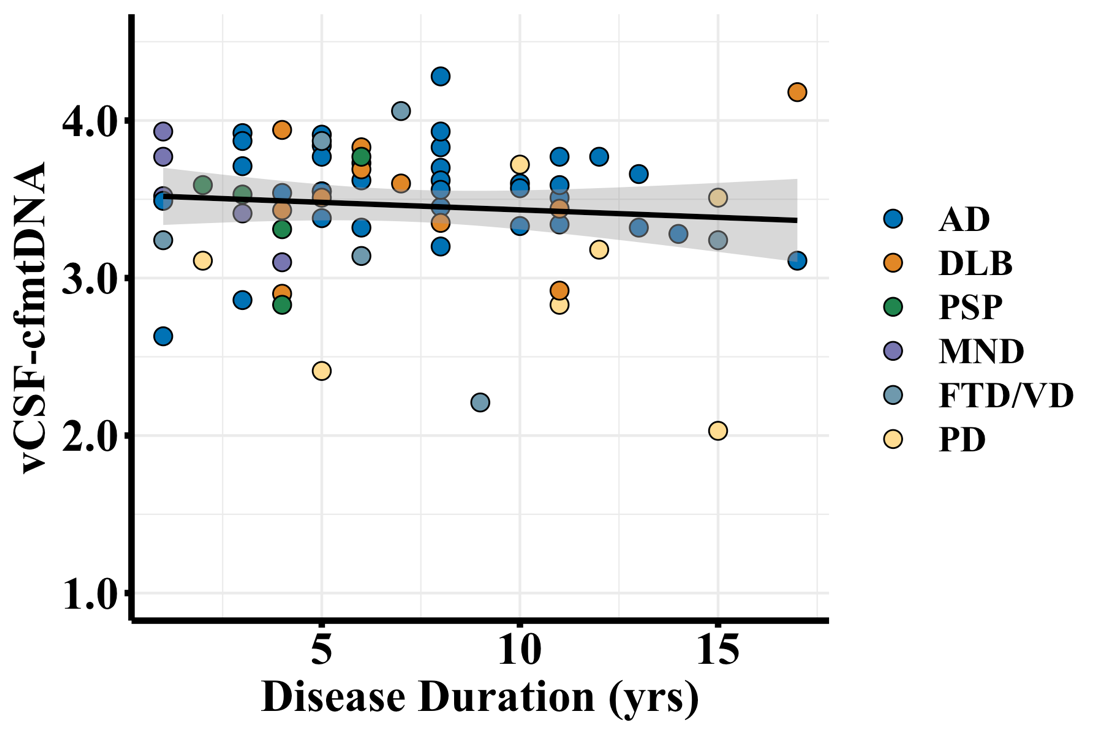
*

***SFigure 3.*** *Scatter plot of NDDs disease duration (x, years) against vCSF-cfmtDNA levels (y). Where, AD=Alzheimer’s Disease, DLB=dementia with Lewy bodies, PSP=progressive supranuclear palsy, MND=motor neuron disease, FTD/VD=frontotemporal lobar dementia/vascular dementia and PD=Parkinson’s disease. and matched controls. Line is linear regression line (r2=<0.2) with 95% confidence interval, p>0.05.*

*
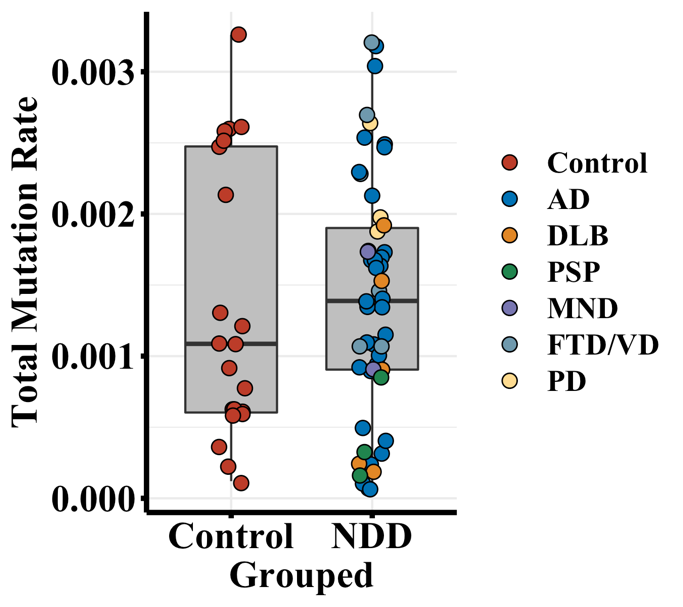

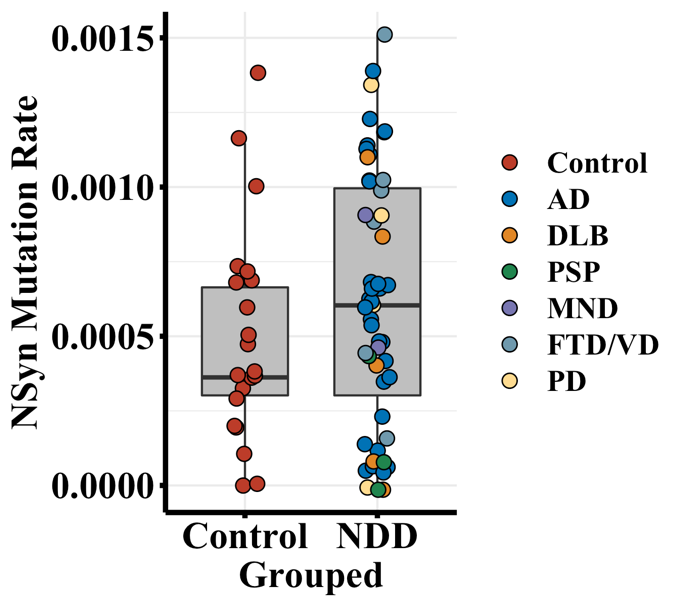

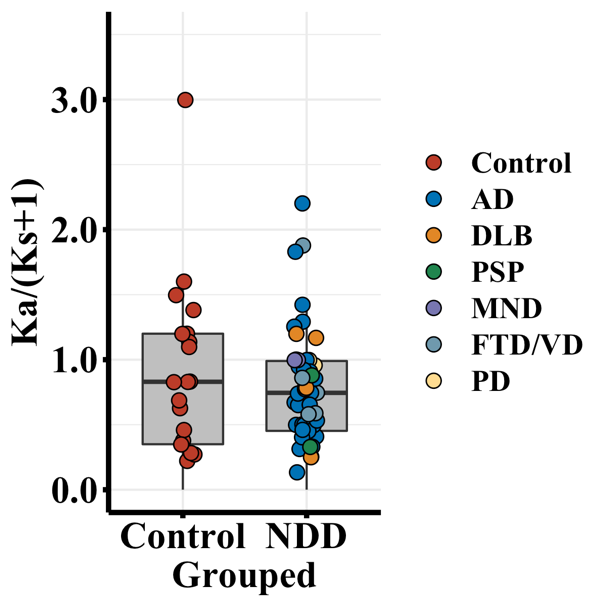
*

***SFigure 4.*** *Boxplots of vCSF-cfmtDNA integrity in NDDs combined, showing the comparative total vCSF-cfmtDNA heteroplasmic mutation rate in combined NDD cases and matched controls* ***(upper left)****, the comparative non-synonymous vCSF-cfmtDNA heteroplasmic mutation rate in combined NDD cases and matched controls* ***(upper Right)*** *and the vCSF-cfmtDNA Ka/Ks(+1) for combined NDD cases and matched controls* ***(lower left)****. Where AD=Alzheimer’s Disease, DLB=dementia with Lewy bodies, PSP=progressive supranuclear palsy, MND=motor neuron disease, FTD/VD=frontotemporal lobar dementia/vascular dementia and PD=Parkinson’s disease. Boxes show median, 25^th^ and 75^th^ percentile and whiskers show 95% confidence interval.*
